# Supplementary material for: The interactome of intact mitochondria by cross-linking mass spectrometry provides evidence for coexisting respiratory supercomplexes
Source: Mol Cell Proteomics. 2017 Dec 8;17(2):216–32. doi: 10.1074/mcp.RA117.000470 (PMC5795388; doi:10.1074/mcp.RA117.000470)
Supplement: Supplemental Data [file supp_RA117.000470_133922_0_supp_23978_fzffwf.zip › spectra_annotation/mito_DR_spectra_annotation/355-1-10-1-2-1.pdf]

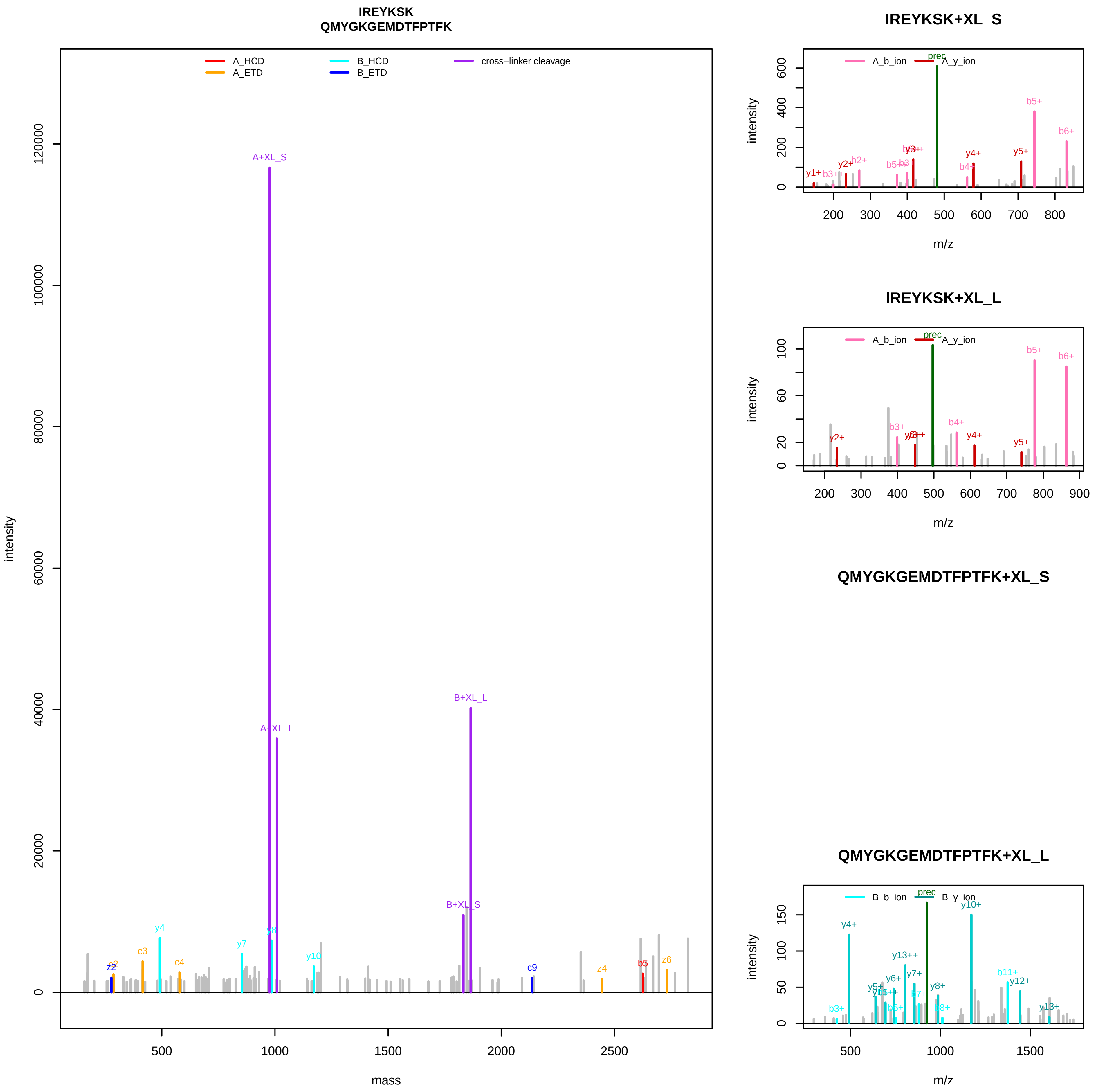
IREYKSK
QMYGKGEMDTFPTFK
A_HCD
A_ETD
B_HCD
B_ETD
cross−linker cleavage
A+XL_S
A+XL_L
B+XL_L
B+XL_S
y4
y7
y8
y10
z2
c3
c4
c9
z4
b5
z6
intensity
mass
IREYKSK+XL_S
IREYKSK+XL_L
QMYGKGEMDTFPTFK+XL_S
QMYGKGEMDTFPTFK+XL_L
A_b_ion
A_y_ion
B_b_ion
B_y_ion
prec
b5+
b6+
y4+
y5+
y2+
b2+
b3+
b4+
y1+
y3+
y10+
y13++
y11++
y6+
y7+
y8+
b11+
y12+
y13+
b3+
b8+
m/z
